# Advances in Molecular Mechanisms of Gastrointestinal Tumors

**DOI:** 10.3390/cancers16081603

**Published:** 2024-04-22

**Authors:** Shihori Tanabe

**Affiliations:** Division of Risk Assessment, Center for Biological Safety and Research, National Institute of Health Sciences, Kawasaki 210-9501, Japan; stanabe@nihs.go.jp; Tel.: +81-44-270-6686

## 1. Introduction

Gastrointestinal cancer is one of the most common malignancies worldwide. The molecular mechanisms of gastrointestinal cancer, particularly several types that are resistant to treatment, have not been fully elucidated. The Special Issue entitled “Advances in Molecular Mechanisms of Gastrointestinal Tumors” includes a collection of a variety of articles on gastrointestinal stromal tumors, colorectal cancer, esophageal squamous cancer, gastrointestinal tumors, gastric carcinogenesis, and gastric cancer. This editorial aims to summarize recent perspectives on the mechanisms of gastrointestinal tumors, where molecular pathway networks are involved.

Epithelial–mesenchymal transition (EMT) is essential to the development of drug resistance in cancer, metastasis, and recurrence of cancer [1]. The microenvironment and EMT are involved in gastrointestinal tumor progression such as metastatic colorectal cancer [2]. Recent findings highlight the importance of molecular mechanisms in terms of microenvironmental and immune regulations in gastrointestinal tumors [3,4,5,6,7]. Chronic inflammation and the gut microbiota, in relation to immune response, have been closely investigated in gastrointestinal tumors [8,9].

Furthermore, phytochemicals have been found to be effective in gastrointestinal cancer, which underscores the importance of understanding the molecular pathway mechanisms regulated by phytochemicals as anti-gastrointestinal tumor agents [10]. The modes of action of phytochemicals include inhibiting pathways related to either wingless-type MMTV integration site family (Wnt)/β-catenin, apoptosis, phosphoinositide 3-kinase (PI3K)/protein kinase B (PKB, AKT)/mammalian target of rapamycin (mTOR), mitogen-activated protein kinase (MAPK), or NF-κB, or otherwise detoxification enzymes or adenosine monophosphate (AMP)-activated protein kinase [10]. It is crucial to reveal the molecular mechanisms of gastrointestinal tumors to develop novel therapeutics to overcome drug resistance.

## 2. An Overview of Published Articles in the Special Issue

The Special Issue “Advances in Molecular Mechanisms of Gastrointestinal Tumors” (https://www.mdpi.com/journal/cancers/special_issues/molecular_gastrointestinal) (accessed on 20 April 2024) was created on 23 November 2021 and the call for submissions of manuscripts was closed on 15 September 2023. Twenty-eight manuscripts were submitted for consideration for this Special Issue, and all of them were subject to the rigorous *Cancers* review process. In total, eleven papers were finally accepted for publication in this Special Issue, including seven articles and four reviews (as of 17 January 2024).

Tan X. et al. focused on the role of CD155 in relation to immunotherapies such as anti-PD-1 and anti-PD-L1 antigens in esophageal squamous cell cancer (ESCA). CD155 is highly expressed in ESCA tissues and is associated with poor patient prognosis. The expression of *CD155* is positively associated with *PD1*, *PDL1*, *CD4*, *IL2RA*, and *S100A9* expression in ESCA. CD155 may be involved in ESCA proliferation.

Proaño-Pérez, E. et al. investigated that the silencing of SH3 Binding Protein 2 (SH3BP2) downregulated KIT, platelet-derived growth factor receptor alpha (PDGFRA), and microphthalmia-associated transcription factor (MITF). It was revealed that SH3BP2 silencing decreased the ETV1 level through miR-1246 and miR-5100, which led to the reduced tumor growth of gastrointestinal stromal tumors (GISTs). The KIT-SH3BP2-MITF/ETV1 pathway may play a role in GIST growth.

Abdul Razzaq E. et al. revealed that overexpression of erb-b2 receptor tyrosine kinase 2 (*ERBB2*) (human epidermal growth factor receptor 2 (HER2)) in colorectal cancer (CRC) is associated with the Wnt signaling pathway in tumorigenesis. HER2 is suggested to be a target for revealing the CRC pathogenesis.

Yu W. et al. highlighted the importance of the enhancer of zeste homolog 2 (EZH2), a catalytic subunit of polycomb repressor complex 2 (PRC2), in gastric cancer (GC). The correlation between the EZH2 gene and gastric carcinogenesis was described, concluding that high expression of EZH2 leads to poor prognosis in GC.

Yan H. et al. focused on G-protein-coupled receptor (GPCR) signaling in GC initiation and progression. GPCR-mediated metastasis and tumor microenvironment remodeling were summarized in terms of their influence on the extracellular matrix, immune cells, stromal cells, sphingosine-1 phosphate receptors, thrombin receptors, and chemokine-chemokine receptors.

Macharia J. et al. revealed that *Aloe secundiflora* extracts have some potential in CRC treatment. The *Aloe secundiflora* methanolic extracts regulated the gene expression of the specific genes in CRC and the rate of apoptosis in Caco-2 colorectal cancer cell lines.

Kamińska, J. et al. focused on the progesterone (P4) and P4 receptor membrane component 1 (PGRMC1)/neuron-derived neurotrophic factor (NENF) complex interactions in CRC. The PGRMC1 and NENF in non-classical P4 signaling may interact as a complex that induces tumor proliferation and invasion.

Cheng, X. et al. investigated the mechanism related to ferroptosis to overcome drug resistance in CRC. Ferroptosis is a unique form of cell death, which is characterized by the iron-dependent accumulation of lipid peroxides. Targeting ferroptosis is a potential therapeutic strategy for CRC.

Shi, J. et al. revealed that synaptotagmin 1 (SYT1) inhibits EMT by negatively regulating ERK/MAPK signaling to suppress CRC cell migration and invasion. It is suggested that SYT1 represses CRC metastasis through blood vessels.

Jovanovic, M. et al. identified the morphological computed tomography features of tumors and the texture analysis parameters. These features represent imaging biomarkers that may be useful for the preoperative prediction of high-risk GISTs.

Aebisher, D. et al. summarized cancer treatment using photodynamic therapy and associated immunological anti-tumor mechanisms in gastrointestinal tumors. Photodynamic therapy is based on oxygen, photosensitizers, and light to induce tumor cell death through the production of reactive oxygen species (ROS).

## 3. Conclusions

In conclusion, the elucidation of the mechanisms of gastrointestinal tumors leads to the progression of advanced therapeutics for cancer. Targeting the components essential in the signaling pathways of gastrointestinal tumors has high potential as therapeutics and diagnostic markers in gastrointestinal tumors.
